# A meta-analysis of controlled clinical trials comparing peginterferon-α plus nucleos(t)ide analogs versus peginterferon-α monotherapy for HBsAg clearance or seroconversion in patients with chronic hepatitis B

**DOI:** 10.3389/fmed.2025.1716225

**Published:** 2026-01-08

**Authors:** Shuyao Zhu, Zhilin Yang, Yuanyuan Wang, Xuebin Cai, Chaochao Qin

**Affiliations:** 1 Department of Clinical Pharmacy, West China Hospital, Sichuan University, Chengdu, Sichuan, China; 2 Oncology Internal Medicine Treatment Unit, Guangyuan Traditional Chinese Medicine Hospital Affiliated to Chengdu University of Traditional Chinese Medicine, Guangyuan, Sichuan, China; 3 Department of Abdominal Surgery, West China Hospital, Sichuan University, Chengdu, Sichuan, China

**Keywords:** chronic hepatitis B, HBsAg seroconversion, HBsAg clearance, nucleos(t)ide analogues, peginterferon

## Abstract

**Background:**

Chronic hepatitis B virus (HBV) infection remains a major global health challenge. Peginterferon-α (PEG-IFN-α) and nucleos(t)ide analogs (NAs) are standard therapies used to suppress HBV replication. Although HBsAg clearance and seroconversion are ideal therapeutic endpoints, whether PEG-IFN-α combined with NAs offers superior efficacy compared with PEG-IFN-α monotherapy remains controversial.

**Methods:**

Controlled clinical trials published between 2000 and 2025 that compared PEG-IFN-α plus NA combination therapy with PEG-IFN-α monotherapy in patients with chronic hepatitis B, with treatment duration ≥ 48 weeks, were included. The primary outcomes were HBsAg clearance and seroconversion.

**Results:**

Eleven trials involving 2,439 patients were identified and analyzed. After 48 weeks of treatment, the HBsAg clearance rate was significantly higher in the combination therapy group than in the monotherapy group [odds ratio (OR) = 1.59, 95% confidence interval (CI): 1.01–2.52, *P* = 0.05]. However, no significant difference in HBsAg clearance rates was observed between the two groups at 24 weeks of post-treatment follow-up (OR = 1.33, 95% CI: 0.76–2.33, *P* = 0.31). Likewise, no significant differences were found in HBsAg seroconversion rates after 48 weeks of treatment or at 24 weeks post-treatment follow-up (OR = 1.77, 95% CI: 0.94–3.32, *P* = 0.08; and OR = 1.42, 95% CI: 0.88–2.28, *P* = 0.15, respectively).

**Conclusion:**

PEG-IFN-α promotes HBsAg clearance and seroconversion. Combination therapy with PEG-IFN-α plus NAs yields higher short-term clearance than monotherapy; however, sustained benefits appear to require prolonged treatment.

## 1 Introduction

Chronic hepatitis B virus (HBV) infection remains a major global public health concern, affecting millions of individuals worldwide (1, 2). HBV reactivation can lead to liver injury and clinically manifests as hepatitis (3, 4). Antiviral therapy is recognized as the first-line treatment for chronic hepatitis B (CHB) (5). Although hepatitis B e-antigen (HBeAg) seroconversion is considered an important treatment endpoint in HBeAg-positive CHB (5, 6), the clearance and seroconversion of hepatitis B surface antigen (HBsAg) represent the outcomes most closely associated with a functional cure (7, 8). Current therapeutic options for CHB primarily include interferon-based therapy and oral nucleos(t)ide analogs (NAs). Peginterferon-α (PEG-IFN-α) is widely used to suppress HBV replication and reduce liver inflammation and fibrosis progression (9, 10). Although both PEG-IFN-α and NAs are established first-line therapies, international guidelines recommend PEG-IFN-α for appropriately selected patients because of its finite treatment duration and potential to induce durable immunologic control (11, 12).

Several clinical trials have examined whether combining PEG-IFN-α with NAs offers superior efficacy compared with PEG-IFN-α monotherapy (8, 13, 14). However, evidence regarding the benefit of combination therapy for enhancing HBsAg clearance or seroconversion remains inconsistent. In line with current international guidelines (e.g., EASL, AASLD), PEG-IFN-α or NAs are recommended as monotherapy for most patients with chronic hepatitis B, rather than as first-line combination therapy, based on considerations of efficacy, safety, and cost-effectiveness. Nevertheless, the potential of combination strategies to improve rates of functional cure remains a subject of ongoing debate among experts. Therefore, in this study, we conducted a systematic review and meta-analysis of controlled clinical trials to evaluate whether combination therapy with PEG-IFN-α plus NAs provides additional benefit over PEG-IFN-α monotherapy in achieving HBsAg clearance or seroconversion in patients with CHB.

## 2 Materials and methods

### 2.1 Literature search and study design

Eligible trials published between January 1995 and March 29, 2025, were identified through systematic searches of the following electronic databases: PubMed/Medline, EMBASE, the Cochrane Library, ClinicalKey and Web of Science databases. Discrepancies in study selection were resolved through discussion among the reviewers. The search strategy used combinations of the following keywords: “HBsAg,” “chronic hepatitis B,” “hepatitis B,” “HBV,” “peginterferon,” and “pegylated interferon.” In addition, the reference lists of relevant articles and conference proceedings were manually searched to identify any additional eligible studies. Studies were categorized into two intervention groups: PEG-IFN-α plus NA combination therapy and PEG-IFN-α monotherapy. Data were extracted according to predefined efficacy outcomes and study characteristics, and any data discrepancies were resolved by consensus among the reviewers. Separate meta-analyses were conducted for each outcome of interest.

### 2.2 Inclusion and exclusion criteria

We included trials that compared PEG-IFN-α plus NA combination therapy with PEG-IFN-α monotherapy therapy in patients with CHB. Two reviewers independently screened and assessed all retrieved records for eligibility. Studies were included if they met all of the following criteria: (1) Published in English; (2) Controlled clinical trial design; (3) Study population consisting of CHB patients; (4) Intervention comparing PEG-IFN-α plus NA combination therapy with PEG-IFN-α monotherapy; (5) Availability of valid outcome data, either directly reported or derivable; (6) In cases of duplicate publications, only the report with the highest methodological quality was included; (7) Reported outcomes included HBsAg clearance or seroconversion.

Studies were excluded if they met any of the following criteria: (1) Compared conventional IFN-α plus NA with conventional IFN-α monotherapy; (2) Preclinical studies involving animals, cell lines, or other non-human models; (3) Included patients with liver transplantation or co-infection with hepatitis C, hepatitis D, or human immunodeficiency virus; (4) Included patients with a history of alcohol or drug abuse, hepatocellular carcinoma, decompensated liver disease, or severe medical or psychiatric illness; (5) Concurrent use of corticosteroids or immunosuppressive agents; (6) Did not report outcomes related to HBsAg clearance or seroconversion; (7) Reported outcomes unclearly or incompletely; (8) Duplicate publications or lack of accessible full text. When necessary, study investigators were contacted to obtain missing data. The methodological quality of each included study was assessed using the Jadad quality scale, with scores ≥ 3 considered high quality (9, 10). The literature search was restricted to studies published in English.

### 2.3 Outcome measures

The primary outcomes were HBsAg clearance and seroconversion. HBsAg clearance was defined as the complete loss of HBsAg from the serum. HBsAg seroconversion was defined as HBsAg loss accompanied by the presence of anti-HBs antibodies. Secondary outcomes included virological and serological responses (HBeAg loss and HBeAg seroconversion), the proportion of patients achieving HBV DNA levels < 400 copies/mL, and biochemical response, defined as normalization of alanine aminotransferase (ALT).

### 2.4 Statistical analysis

This meta-analysis evaluated differences in treatment efficacy between PEG-IFN-α monotherapy and PEG-IFN-α plus NA combination therapy in CHB patients. Statistical analyses and forest plot construction were performed using Review Manager (RevMan) software (version 5.4.1) (15). Pooled outcomes included HBsAg clearance and seroconversion, HBeAg clearance and seroconversion, the proportion of patients receiving HBV DNA < 400 copies/mL, and ALT normalization at 48 and 72 weeks. Effect sizes were summarized using odds ratio (OR) with corresponding 95% confidence interval (95% CI). Fixed- or random-effects models were selected according to the level of heterogeneity.

Statistical heterogeneity across studies was assessed using the Chi-square test and quantified with the I^2^ statistic, which ranges from 0 to 100%. Heterogeneity was considered significant when *P* < 0.10. A fixed-effects model was applied when heterogeneity was low (*I*^2^ < 50%, *P* ≥ 0.05); otherwise, a random-effect model was used (*I*^2^ ≥ 50%, *P* < 0.05).

Potential publication bias for the primary outcomes was evaluated using funnel plots and further examined with Egger’s and Begg’s tests using StataMP (version 18.0). The risk of bias for each included study was assessed and categorized as low, high, or unclear according to established methodological criteria (16–18).

## 3 Results

### 3.1 Study selection and characteristics

**FIGURE 1:**
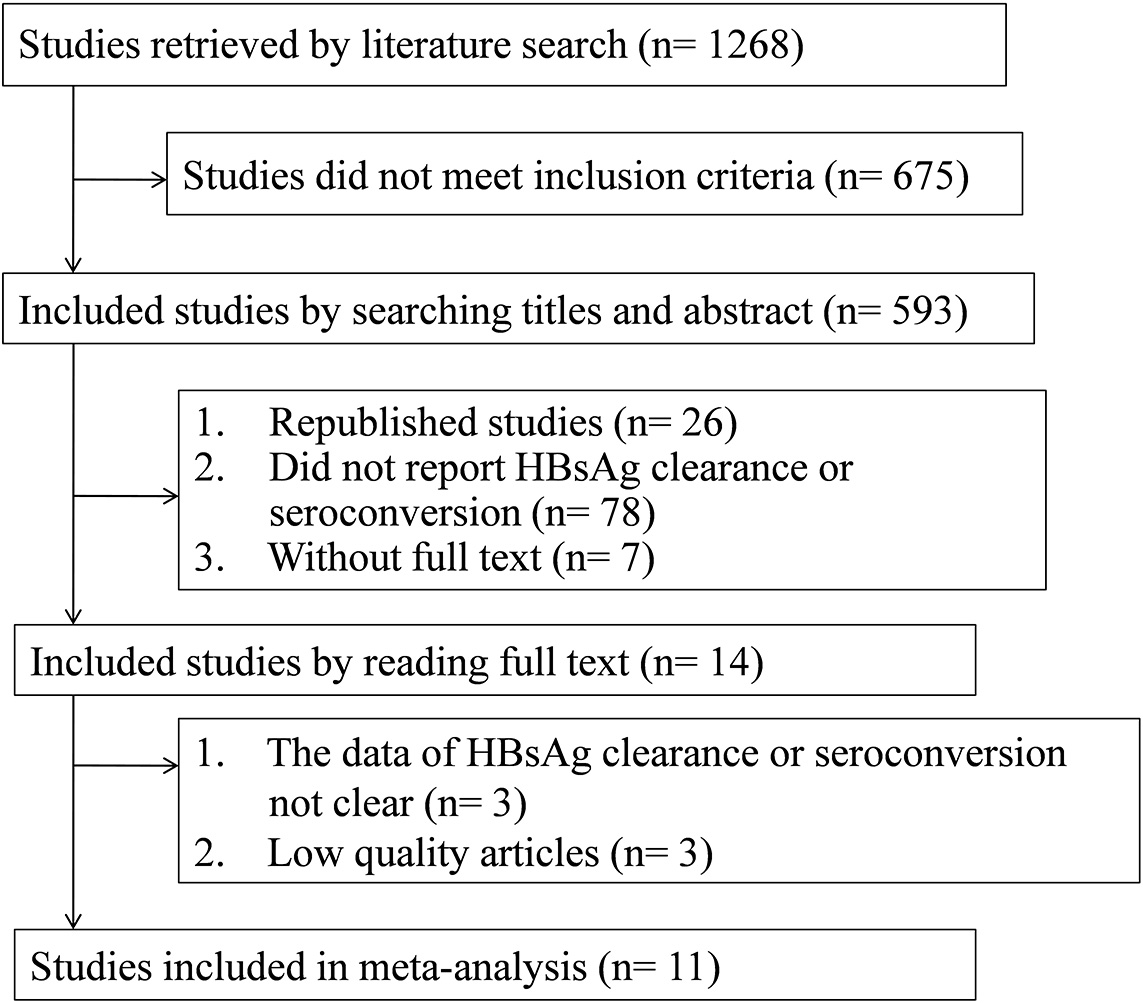
Literature search and data extraction.

The study selection process is illustrated in Figure 1. A total of 1,268 articles were initially identified. Of these, 593 potentially relevant controlled clinical trials evaluating PEG-IFN-α-based therapy for chronic HBV infection underwent detailed screening. After removing 26 duplicate records, 85 studies were excluded based on a lack of full text or irrelevance according to titles and abstracts. Of the remaining 14 articles, 3 were excluded because they did not clearly report HBsAg clearance or seroconversion outcomes or their methodological quality was insufficient for inclusion in a meta-analysis. Ultimately, 11 studies met all predefined criteria and were included in the final analysis. Table 1 summarizes the main characteristics of the included studies, which collectively enrolled 2,758 patients. Nine studies (involving 2,546 patients) were multicenter, double-blind controlled trials. Based on the Jadad scale, seven randomized controlled trials (RCTs) scored between 4 and 6 points, indicating high methodological quality; the remaining two non-RCTs also scored 4-6. Among the included studies, four reported both HBsAg clearance and seroconversion outcomes (13, 14, 19–22), three reported only HBsAg clearance (23–25), and two reported only HBsAg seroconversion (26, 27). All patients received treatment for 48 weeks, followed by a 24-week post-treatment follow-up period. Ten studies evaluated concurrent combination therapy, while one study assessed sequential therapy (23). All studies were available as full-text publications in English.

**TABLE 1:** Characteristics of the studies included in the meta-analysis.

Study (year)	Study type	Treatment type	n	Treatment period	Follow-up period	Jadad score	Patient characteristics	Naive or experienced	NA type	Post-treatment hepatic flare reported
Boglione L., 2013 (20)	Non-RCT	PEG-IFN-α PEG-IFN-α plus NA	40	48 weeks	24 weeks	4	HBeAg +, high HBV DNA, non-D genotypes, adults	Naive	Entecavir	Not specified
Bunyamin D., 2004 (27)	RCT	PEG-IFN-α PEG-IFN-α plus NA	182	48 weeks	24 weeks	6	Children, HBeAg +, elevated ALT, CHB	Naive	Lamivudine	Moderate flare only in monotherapy group
Erik H., 2008 (23)	Non-RCT	PEG-IFN-α PEG-IFN-α plus NA	172	48 weeks	24 weeks	5	HBeAg +, adults, mixed genotypes, from prior RCT	Both (prior study included naive and experienced)	Lamivudine	Not specified
George K., 2005 (26)	RCT	PEG-IFN-α PEG-IFN-α plus NA	542	48 weeks	24 weeks	5	HBeAg +, mostly Asian, adults, high HBV DNA	Both (prior therapy allowed)	Lamivudine	ALT elevations reported, no significant difference between groups
Harry L., 2005 (19)	RCT	PEG-IFN-α PEG-IFN-α plus NA	266	48 weeks	24 weeks	5	HBeAg +, adults, mixed genotypes, elevated ALT	Both (21% IFN-experienced)	Lamivudine	Hepatitis flare reported as serious adverse event in both groups
Marcellin P., 2004 (22)	RCT	PEG-IFN-α PEG-IFN-α plus NA	356	48 weeks	24 weeks	5	HBeAg-negative, adults, mixed race, bridging fibrosis/cirrhosis ∼27%	Both (prior lamivudine 4–8%, prior interferon 6–10%)	Lamivudine	ALT elevations during and after treatment reported
Marcellin P., 2016 (21)	RCT	PEG-IFN-α PEG-IFN-α plus NA	555	48 weeks	24 weeks	5	HBeAg-positive and negative, non-cirrhotic, adults, mixed genotypes	Naive for interferon and nucleotide analogs (prior nucleoside allowed if stopped > 24 weeks)	Tenofovir disoproxil fumarate	ALT elevations and hepatic flares reported, with details
Martijn J., 2006 (24)	RCT	PEG-IFN-α PEG-IFN-α plus NA	266	48 weeks	24 weeks	5	HBeAg +, adults, mixed genotypes, elevated ALT	Both (21% IFN-experienced, 12% lamivudine-experienced)	Lamivudine	ALT flares reported, but not specifically post-treatment
Paola P., 2009 (25)	RCT	PEG-IFN-α PEG-IFN-α plus NA	60	48 weeks	24 weeks	4	HBeAg-negative, compensated CHB, median age 48, 67% male, mean ALT 3.3x ULN, HBV DNA 5.8 log10 IU/mL	Both (prior IFN 8.3%, prior NA 18.3%)	Adefovir dipivoxil	Not specifically reported
Jiang-Shan Lian, 2022 (14)	RCT	PEG-IFN-α PEG-IFN-α plus NA	181	48 weeks	24 weeks	6	Details not fully available (assumed HBeAg-positive or negative adults)	Not specified	Not specified	Not specified
Yan Xu 2017 (13)	RCT	PEG-IFN-α PEG-IFN-α plus NA	138	48 weeks	24 weeks	6	Details not fully available (assumed HBeAg-positive or negative adults)	Not specified	Not specified	Not specified

### 3.2 Primary outcomes: HBsAg clearance and seroconversion with PEG-IFN-α plus NA combination therapy versus PEG-IFN-α monotherapy

**FIGURE 2:**
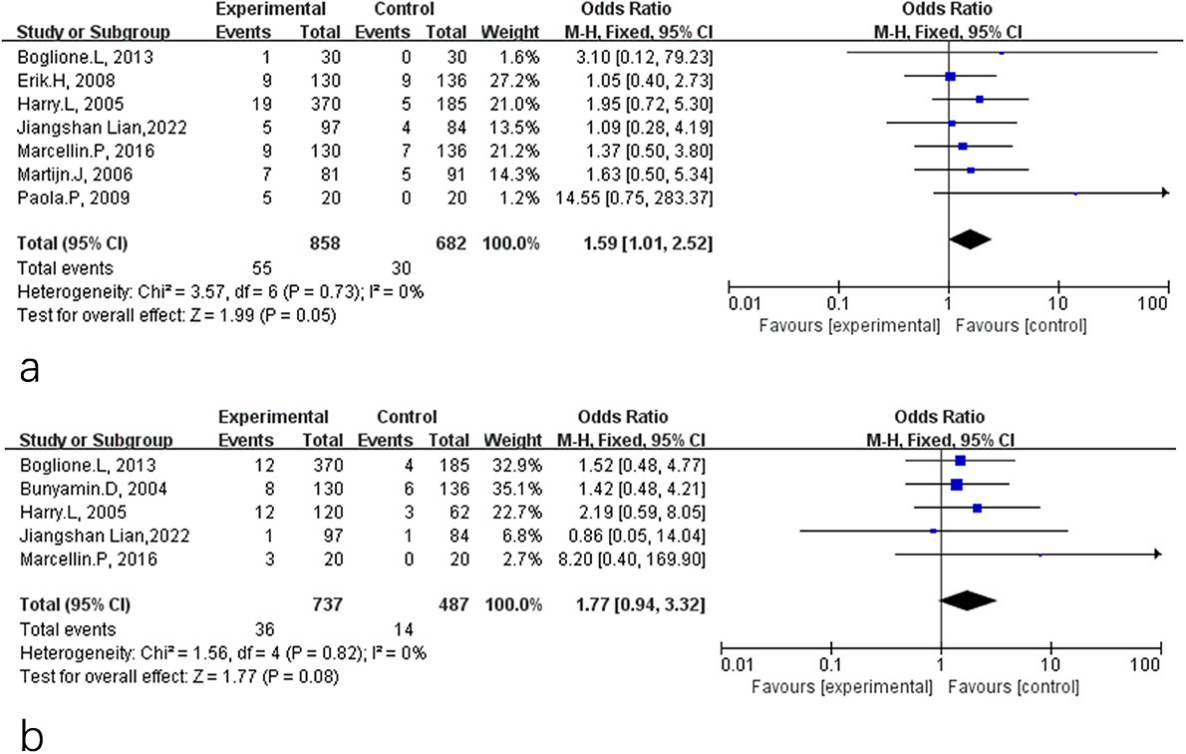
Analysis of HBsAg clearance **(a)** and HBsAg seroconversion **(b)** between PEG-IFN-α+NA combination and PEG-IFN-α monotherapy after 48 weeks treatment (overall trials).

At the end of treatment, the rates of HBsAg clearance and seroconversion were 6.4 and 4.9%, respectively, in the PEG-IFN-α plus NA combination group, compared to 4.4 and 2.9% in the PEG-IFN-α monotherapy group. The corresponding forest plots are shown in Figure 2. Because heterogeneity was low, all analyses applied a fixed-effect model. Pooled analysis of seven studies demonstrated that comparison therapy significantly increased the HBsAg clearance rate after 48 weeks of treatment compared to monotherapy (OR = 1.59, 95% CI: 1.01–2.52, *P* = 0.05, *I*^2^ = 0%) (Figure 2a). In contrast, no significant differences was observed in HBsAg seroconversion at week 48 (OR = 1.77, 95% CI: 0.94–3.32, *P* = 0.08, *I*^2^ = 0%) (Figure 2b).

**FIGURE 3:**
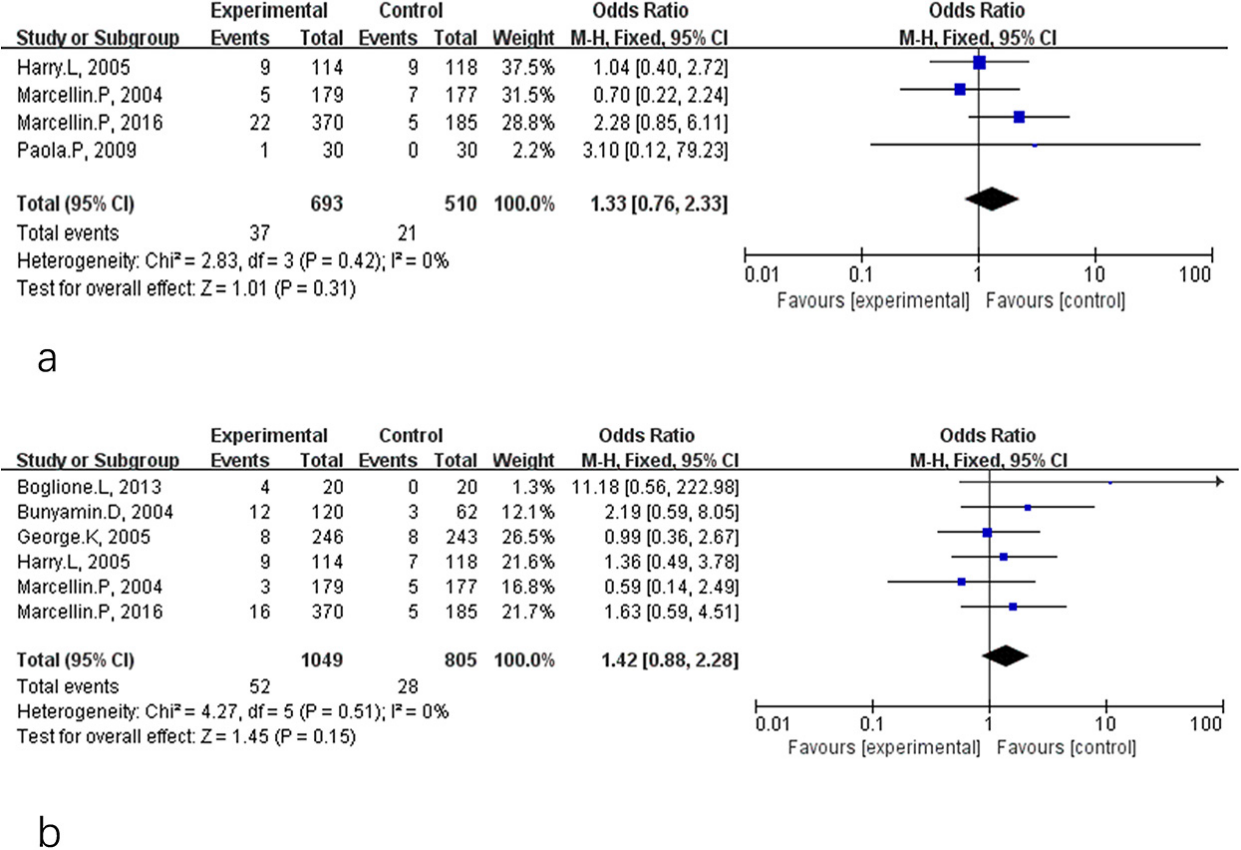
Analysis of HBsAg clearance **(a)** and HBsAg seroconversion **(b)** between PEG-IFN-α+NA combination and PEG-IFN-α monotherapy after 24 weeks follow-up (overall trials).

A fixed-effect model was similarly used for the 24-week post-treatment follow-up analyses. During follow-up, the rates of HBsAg clearance and seroconversion were 5.3 and 5.0% in the combination therapy group and 4.1 and 3.5% in the monotherapy group, respectively. Pooled analysis of four studies showed no significant difference in HBsAg clearance rates at 24 weeks post-treatment (OR = 1.33, 95% CI: 0.76–2.33, *P* = 0.31, *I*^2^ = 0%) (Figure 3a) or in HBsAg seroconversion (OR = 1.42, 95% CI: 0.88–2.28, *P* = 0.15, *I*^2^ = 0%) (Figure 3b).

### 3.3 Secondary outcomes: HBeAg serological response

**FIGURE 4:**
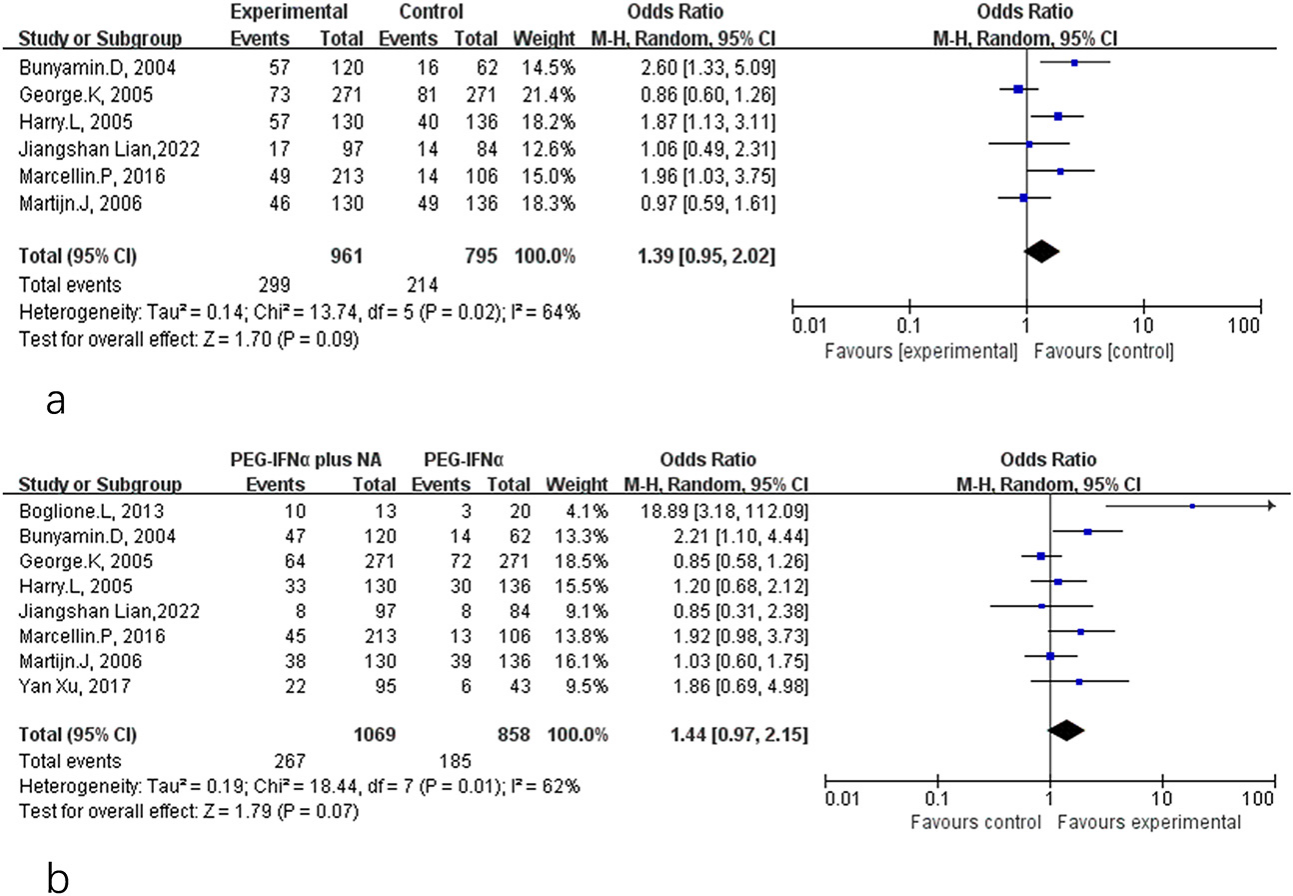
Analysis of HBeAg clearance **(a)** and HBeAg seroconversion **(b)** between PEG-IFN-α+NA combination and PEG-IFN-α monotherapy after 48 weeks treatment (overall trials).

**FIGURE 5:**
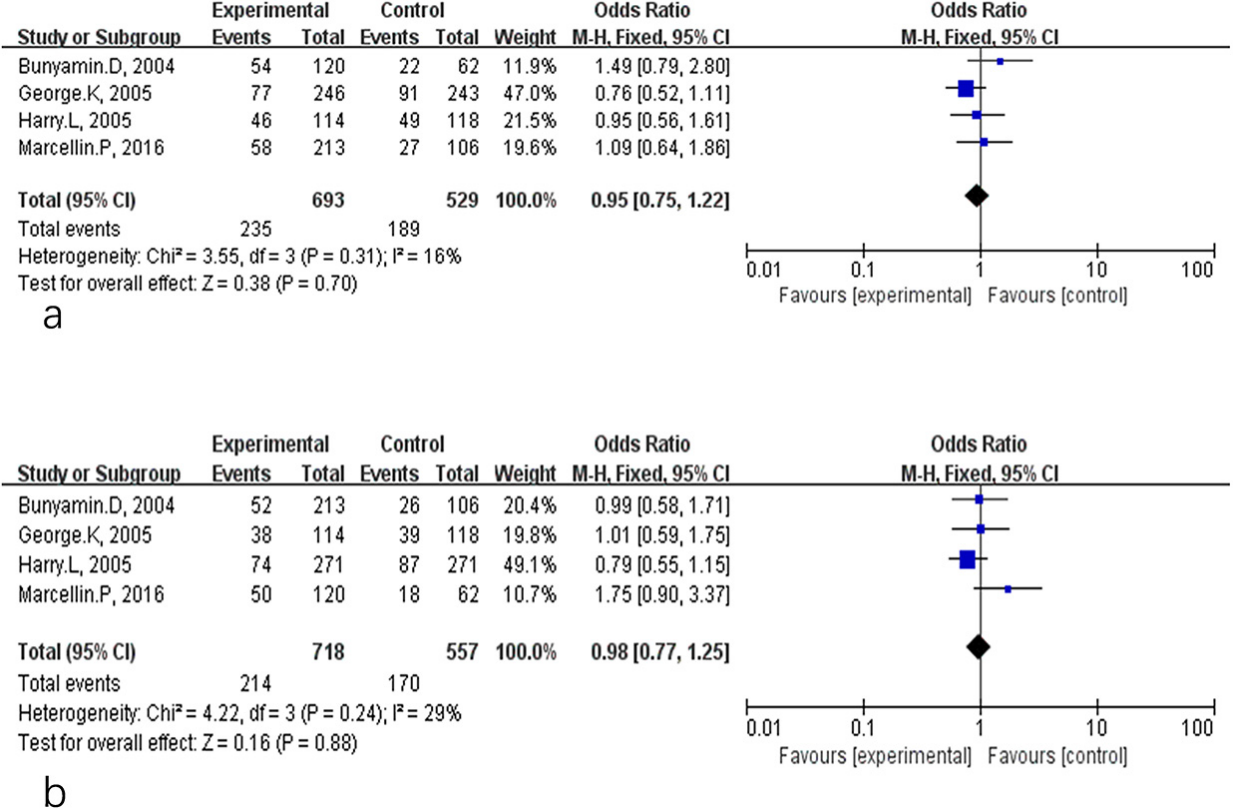
Analysis of HBeAg clearance **(a)** and HBeAg seroconversion **(b)** between PEG-IFN-α+NA combination and PEG-IFN-α monotherapy after 24 weeks follow-up (overall trials).

HBeAg serological response included both HBeAg loss and HBeAg seroconversion in CHB patients treated with PEG-IFN-α plus NA combination therapy or PEG-IFN-α monotherapy. Comparative analyses at different time points are shown in Figures 4, 5. As presented in Figure 4, there were no significant differences between the combination therapy and monotherapy groups in HBeAg loss after 48 weeks of treatment (OR = 1.39, 95% CI: 0.95–2.02, *P* = 0.09, *I*^2^ = 64%) (Figure 4a). Similarly, no significant difference was observed for HBeAg seroconversion at week 48 (OR = 1.44, 95% CI: 0.97–2.15, *P* = 0.07, *I*^2^ = 62%) (Figure 4b). At the 24-week post-treatment follow-up (Figure 5), the two groups also showed no significant differences in HBeAg loss (OR = 0.95, 95% CI: 0.75–1.22, *P* = 0.70, *I*^2^ = 16%) (Figure 5a) or in HBsAg seroconversion (OR = 0.98, 95% CI: 0.77–1.25, *P* = 0.88, *I*^2^ = 29%) (Figure 5b).

### 3.4 Secondary outcomes: HBV DNA < 400 copies/mL

**FIGURE 6:**
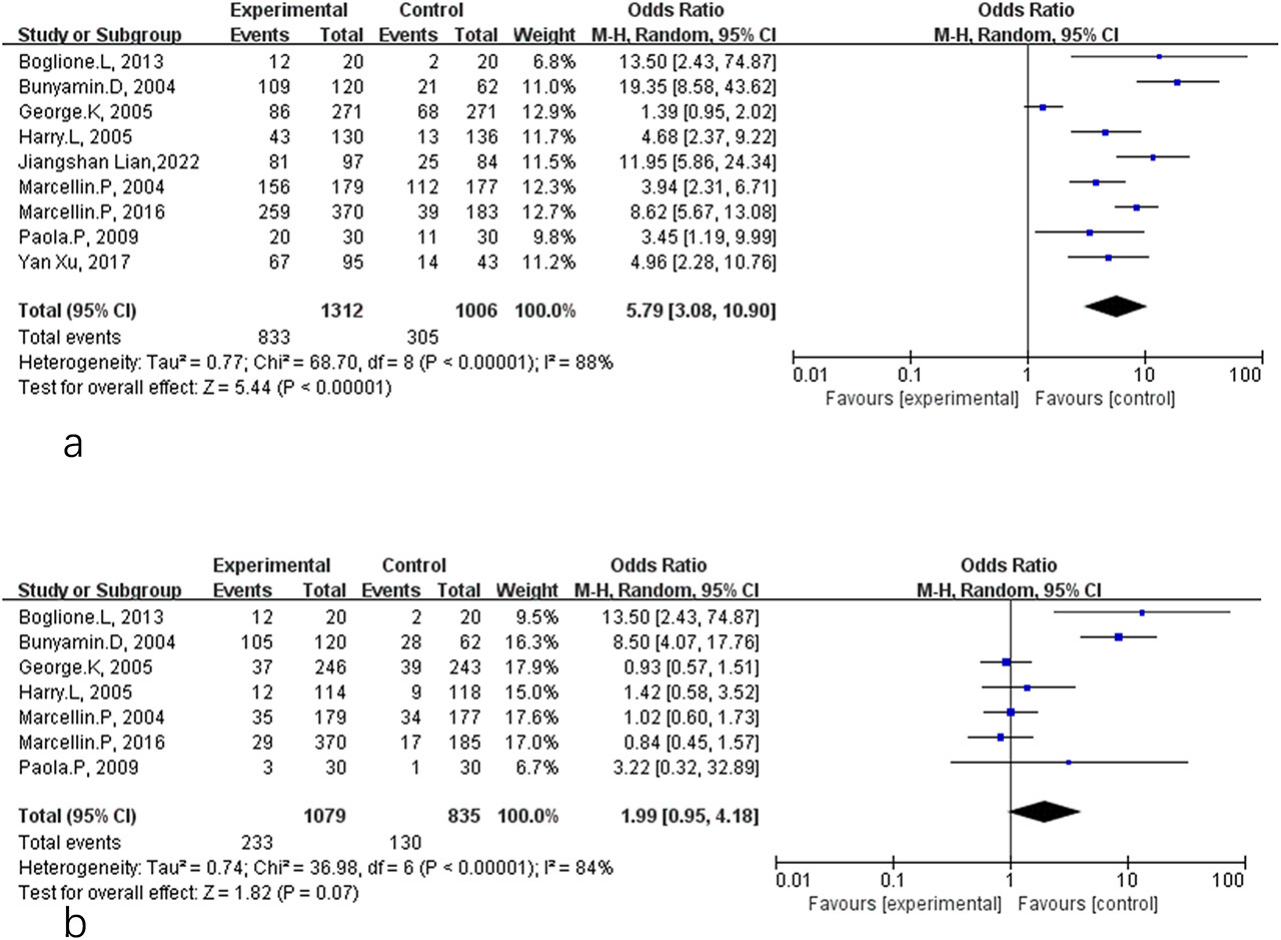
Analysis of HBV DNA < 400 copies/mL rate between PEG-IFN-α+NA combination and PEG-IFN-α monotherapy after 48 weeks treatment **(a)** and after 24 weeks follow-up (overall trials) **(b)**.

Figure 6 compares the proportion of patients achieving HBV DNA < 400 copies/mL in the two treatment groups. A significant difference favoring combination therapy was observed at the end of treatment (OR = 5.79, 95% CI: 3.08–10.90, *P* < 0.01, *I*^2^ = 88%) (Figure 6a). However, this benefit was not sustained during the 24-week follow-up period, and no significant difference was found between groups (OR = 1.99, 95% CI: 0.95–4.18, *P* = 0.07, *I*^2^ = 84%) (Figure 6b).

### 3.5 Secondary outcomes: biochemical response (ALT normalization)

**FIGURE 7:**
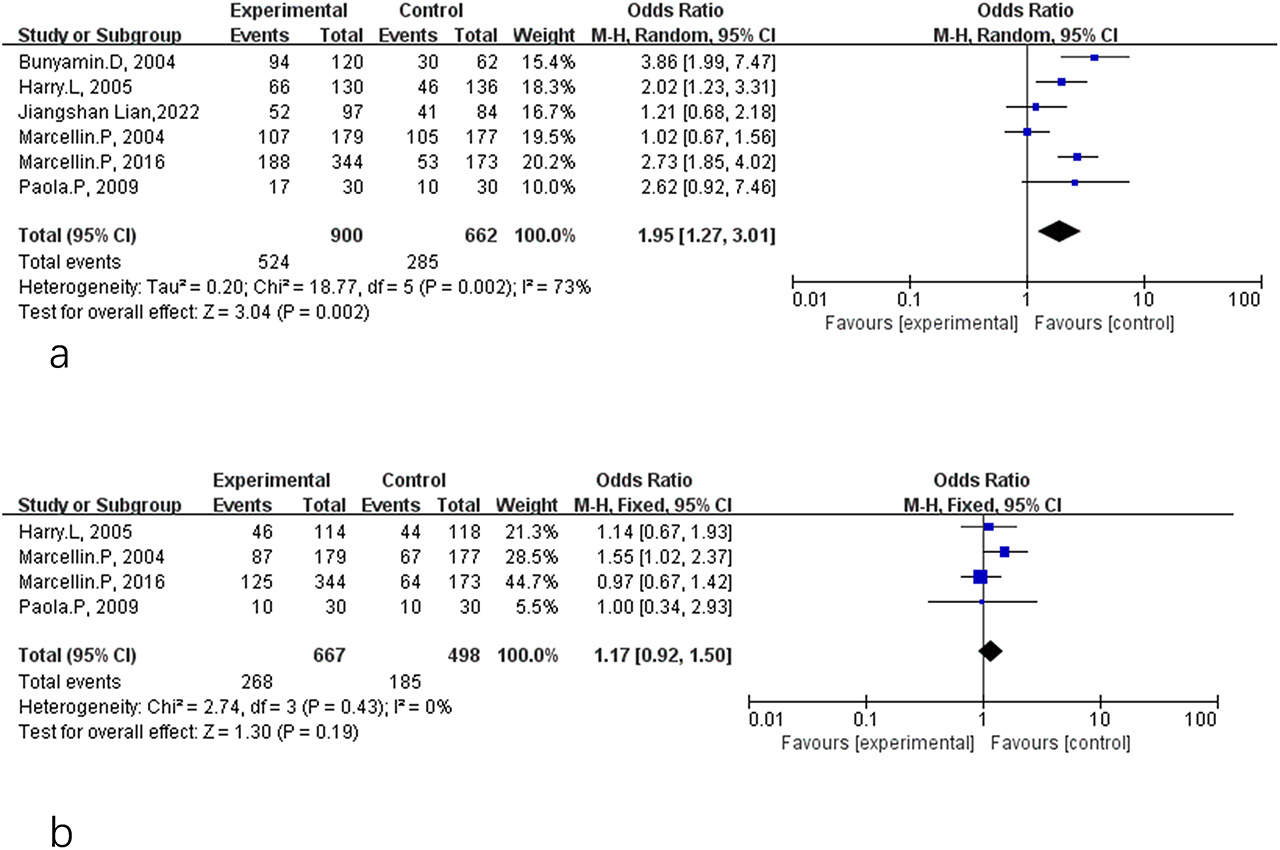
Analysis of ALT normalization rate between PEG-IFN-α+NA combination and PEG-IFN-α monotherapy after 48 weeks treatment (overall trials) **(a)** and after 24 weeks follow-up (overall trials) **(b)**.

Figure 7 summarizes the biochemical response, defined as ALT normalization. After 48 weeks of treatment, combination therapy was associated with a significantly higher frequency of ALT normalization compared with monotherapy (OR = 1.95, 95% CI: 1.27–3.01, *P* < 0.01, *I*^2^ = 73%) (Figure 7a). However, the difference was no longer significant at the 24-week follow-up (OR = 1.17, 95% CI: 0.92–1.50, *P* = 0.19, *I*^2^ = 0%) (Figure 7b).

### 3.6 Publication bias

**FIGURE 8:**
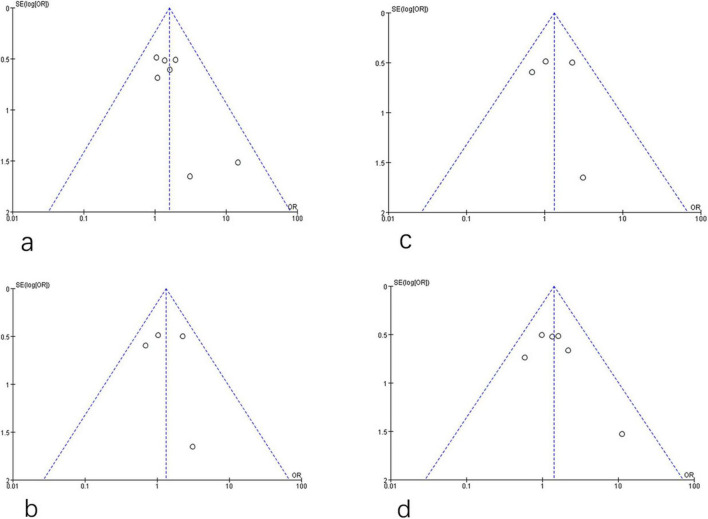
Assessment of publication bias for PEG-IFN-α+NA combination versus PEG-IFN-α monotherapy on HBsAg clearance and HBsAg seroconversion after 48 weeks treatment **(a,b)**, and after 24 weeks follow-up (overall trials) **(c,d)**.

**FIGURE 9:**
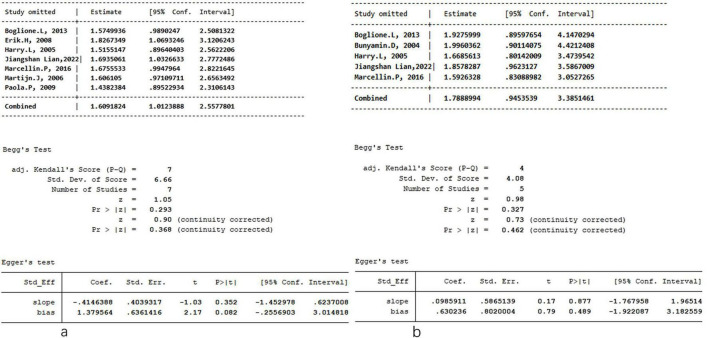
Sensitivity analysis, Egger’s test and Begg’s test to examine the risk of primary outcome potential publication bias for PEG-IFN-α+NA combination versus PEG-IFN-α monotherapy on HBsAg clearance **(a)** and HBsAg seroconversion **(b)** after 48 weeks treatment.

**FIGURE 10:**
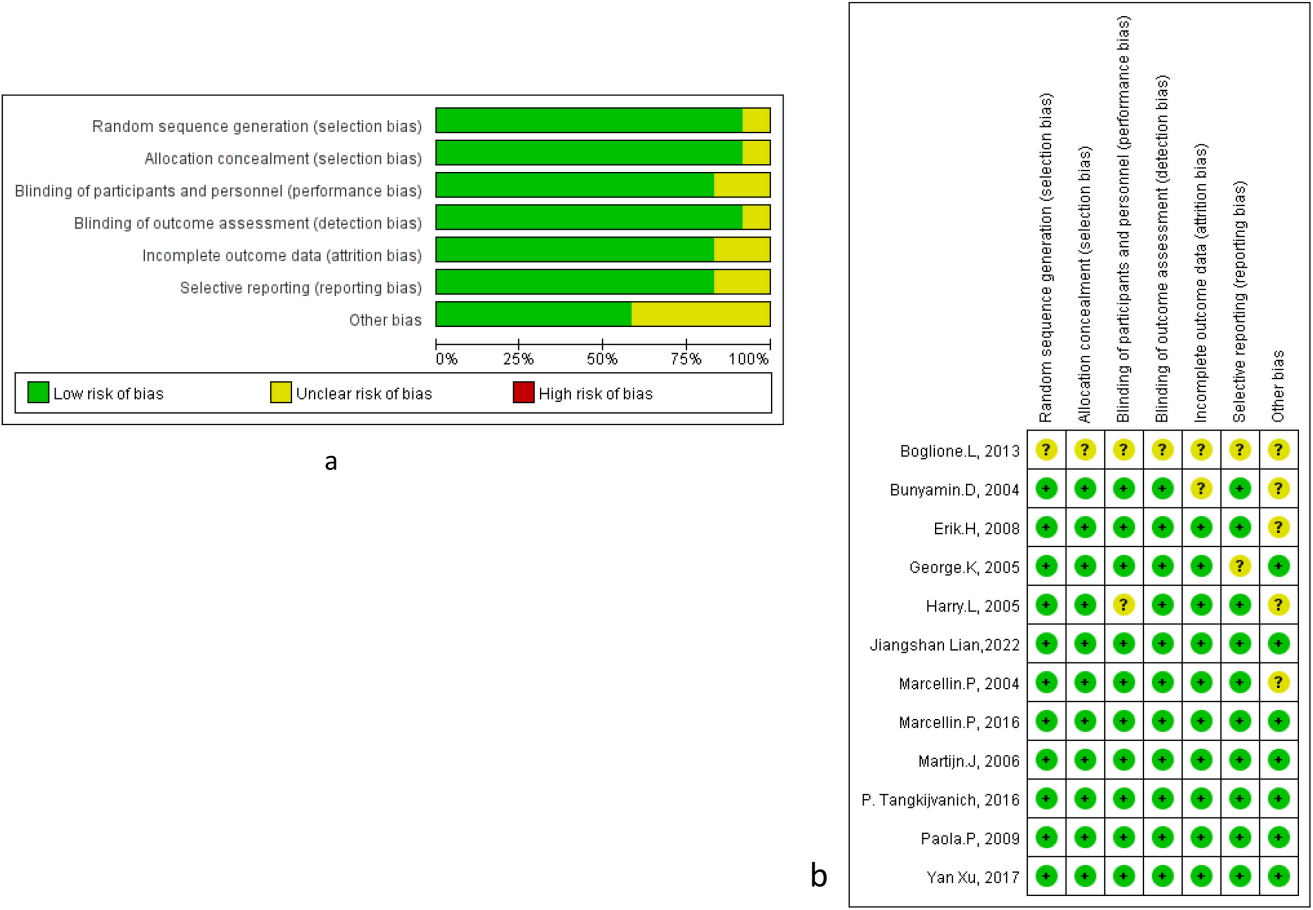
Risk of bias in all included studies was assessed using the Cochrane Collaboration’s tool. **(a)** Risk of bias graph: each risk of bias item was presented as percentages in all included studies. **(b)** Risk of bias summary: each risk of bias item was presented in each included study.

Potential publication bias was assessed using funnel plots for the primary outcomes. No significant publication bias was detected for HBsAg clearance and seroconversion when comparing PEG-IFN-α plus NA combination therapy to PEG-IFN-α monotherapy. The funnel plots showed no appreciable asymmetry for HBsAg clearance (Figure 8a) or HBsAg seroconversion (Figure 8b) at week 48. Similarly, no evidence of publication bias was observed for HBsAg clearance (Figure 8c) or HBsAg seroconversion (Figure 8d) at the 24-week follow-up. Further quantitative assessment confirmed these findings. For HBsAg clearance at week 48, Begg’s test (*P* = 0.368) and Egger’s test (*P* = 0.082) indicated no significant publication bias (Figure 9a). For HBsAg seroconversion, Begg’s test (*P* = 0.462) and Egger’s test (*P* = 0.489) also showed no evidence of significant bias (Figure 9b). A leave-one-out sensitivity analysis was conducted to evaluate the robustness of the pooled estimates for HBsAg clearance (Figure 9a) and seroconversion (Figure 9b) at week 48. Removal of any single study did not materially alter the overall effect estimates, demonstrating that the primary findings of this meta-analysis are stable and reliable. The overall risk of bias for the included studies, evaluated using the Cochrane Collaboration’s risk of bias tool, is presented in Figure 10.

## 4 Discussion

Our study includes several high-quality trials comparing PEG-IFN-α plus NA combination therapy with PEG-IFN-α monotherapy for the treatment of CHB, with a primary focus on HBsAg clearance and seroconversion. By pooling data from all relevant clinical controlled trials, this meta-analysis aims to clarify the ongoing debate regarding the optimal antiviral treatment strategy. However, the limited number of well-controlled trials remains a constraint for drawing definitive conclusions. Numerous studies have evaluated both PEG-IFN-α plus NA combination therapy and PEG-IFN-α monotherapy for their ability to prevent HBV reactivation and achieve virological, serological, and biochemical responses (19–27). Although both regimens demonstrate clinical efficacy, their comparative effectiveness remains uncertain.

Mechanistically, NAs act primarily as HBV DNA polymerase inhibitors (28, 29), whereas PEG-IFN-α suppresses HBV replication by promoting post-transcriptional degradation of HBV RNA and inducing the expression of antiviral proteins (24, 30, 31). NAs generally require long-term, sometimes indefinite, administration to maintain viral suppression, which carries a risk of antiviral resistance (32). In contrast, PEG-IFN-α therapy involves inconvenient subcutaneous administration and is associated with significant adverse effects (33). Given these limitations, combining PEG-IFN-α with an NA is recommended in clinical guidelines for CHB management (19, 20), as this strategy may enhance antiviral efficacy and reduce the risk of drug resistance by targeting HBV through complementary mechanisms (21, 23). Several studies have reported that combination therapy provides superior virological and biochemical responses compared to monotherapy. Some RCTs have demonstrated that combination therapy can achieve greater reductions in HBV DNA levels (24, 27), and higher rates of HBsAg and HBeAg clearance compared to PEG-IFN-α monotherapy (20, 23). However, other clinical trials have reported comparable therapeutic outcomes between the two regimens (19, 20, 27). Contributing to ongoing debate over whether combination therapy offers a meaningful advantage in routine clinical practice. To address this controversy and provide more precise estimates of treatment effects, we conducted this meta-analysis to systematically compare the efficacy of PEG-IFN-α alone versus PEG-IFN-α plus NA combination therapy in patients with CHB.

In our analysis of the primary outcomes, PEG-IFN-α plus NA combination therapy resulted in significantly higher HBsAg clearance rates compared to PEG-IFN-α monotherapy (*P* = 0.05) after 48 weeks of treatment. However, no significant differences were observed between the two groups in terms of HBsAg seroconversion after 48 weeks and HBsAg clearance or seroconversion at 24 weeks post-treatment follow-up (20–22). Regarding secondary outcomes, no significant differences were identified in serological responses, including HBeAg loss and HBeAg seroconversion, between the combination and monotherapy groups (24). Notably, patients receiving PEG-IFN-α plus NA achieved significantly greater HBV DNA suppression (*P* < 0.01) and higher rates of ALT normalization (*P* < 0.01) after 48 weeks of treatment compared to those receiving PEG-IFN-α monotherapy (13). We acknowledge the substantial heterogeneity observed for this outcome (*I*^2^ = 88%). Major sources likely include variability in patient populations, differences in combination treatment regimens, and inconsistencies in assay sensitivity across studies. These factors collectively limit the interpretability and generalizability of the effect-pooled estimate. Taken together, these findings indicate that PEG-IFN-α plus NA combination therapy provides a modest but clinically meaningful advantage in CHB patients in terms of HBsAg clearance, virological response (HBV DNA < 400 copies/mL), and biochemical response (ALT normalization). This suggests that combination therapy may be a preferable option for selected patients. Overall, our results support the notion that combining PEG-IFN-α with an NA enhances HBsAg clearance, ALT normalization, and the control of both clinical and virological HBV reactivation.

The improved outcomes with combination therapy likely reflect the complementary mechanisms of action of the two agents. PEG-IFN-α enhances host immune responses against HBV, while nucleos(t)ide analogs provide sustained suppression of viral replication (21, 27). This dual action may promote virus-specific immune responses, leading to higher rates of HBsAg clearance, biochemical normalization, and virological control. However, it is important to note that only a limited proportion of patients maintained these benefits after treatment cessation (21). In our analysis, patients receiving PEG-IFN-α plus NA for 48 weeks achieved greater reductions in HBV DNA levels and higher ALT normalization rates compared to PEG-IFN-α monotherapy. Whether extending combination therapy beyond 48 weeks could further improve sustained HBV DNA suppression remains an open question and warrants evaluation in future studies (25). Nevertheless, our long-term follow-up analyses did not reveal significant differences in HBsAg clearance or seroconversion, HBeAg clearance or seroconversion, or HBV DNA suppression between the two groups after treatment discontinuation. For instance, at 24 weeks post-treatment, the HBsAg clearance rate among patients receiving PEG-IFN-α plus NA was not significantly higher than among those receiving PEG-IFN-α monotherapy (20). This suggests that while combination therapy may increase HBsAg clearance and ALT normalization at the end of treatment, sustaining these benefits long-term remains challenging. By the end of 48 weeks, patients treated with the combination regimen achieved higher rates of HBsAg seroconversion compared to monotherapy (27), likely reflecting the distinct antiviral mechanisms of the two agents. However, no significant differences were observed in ALT normalization, HBsAg loss, or HBV DNA clearance rates at 24 weeks post-treatment. One potential explanation for this virological “breakthrough” is premature treatment discontinuation, highlighting the need for further investigation in larger, long-term RCTs. For example, in a multicenter randomized trial conducted by Paola Piccolo et al., only one patient (3.3%) in the combination therapy group achieved HBsAg clearance at 24 weeks post-treatment (25).

Overall, our meta-analysis provides direct evidence that PEG-IFN-α plus NA combination therapy, through complementary mechanisms, produces significantly higher rates of HBsAg loss compared to PEG-IFN-α monotherapy after 48 weeks of treatment. Additionally, the combination improves ALT normalization and HBV DNA suppression. Nevertheless, no significant differences were observed in serological outcomes, including HBeAg loss and seroconversion, between the two groups at any time point. These findings suggest that, although combination therapy can improve certain virological and biochemical endpoints, PEG-IFN-α monotherapy remains an effective option, particularly in settings where cost considerations are important. Our analysis demonstrated that the combination regimen significantly improved HBsAg clearance at 48 weeks; however, this advantage was not sustained during follow-up. Whether extending PEG-IFN-α plus NA therapy could further enhance functional cure rates remains uncertain and should be explored in future longer-duration trials. Any incremental benefit from prolonged treatment must also be balanced against the increased cost, adverse effects, and overall treatment burden.

Importantly, the therapeutic benefit of PEG-IFN-α is not uniform across all patient populations. Growing evidence indicates that its addition is most effective in selected subgroups, particularly in individuals with low baseline HBsAg levels. This includes patients receiving long-term NA therapy who have achieved virological suppression but remain uncured. For these individuals, adding or switching to PEG-IFN-α may help overcome immune tolerance and increase the likelihood of HBsAg loss or seroconversion. This meta-analysis has several limitations. Significant heterogeneity existed across studies, resulting from differences in patient characteristics, NA regimens, and study designs, which prevented more refined subgroup analyses, including evaluation of hepatic flare. The absence of individual patient data, especially baseline HBsAg levels, restricted assessment of key predictors of response. Furthermore, the uniform 48-week treatment duration and relatively short follow-up period limit conclusions regarding long-term outcomes and the efficacy of *de novo* combination therapy.

## Data Availability

The original contributions presented in this study are included in this article/supplementary material, further inquiries can be directed to the corresponding author.
